# Editorial: Advances in viticulture: new approaches towards the vineyard of the future

**DOI:** 10.3389/fpls.2024.1475437

**Published:** 2024-08-29

**Authors:** Gastón Gutiérrez-Gamboa, Nicolás Verdugo-Vásquez

**Affiliations:** ^1^ Instituto de Investigaciones Agropecuarias, INIA Carillanca, Vilcún, La Araucanía, Chile; ^2^ Instituto de Investigaciones Agropecuarias, INIA Intihuasi, Vicuña, Coquimbo, Chile

**Keywords:** berry metabolism, climatic variability, genetic diversity, non-destructive technologies, organic soil amendments, soil restoration, vineyard adaptation

Temperatures rising over time are changing weather patterns, altering the balance of the natural cycles of life. Climate change causes an increase in the occurrence of many extreme weather events, which contribute to climate variability. The El Niño–Southern Oscillation (ENSO) is a climate phenomenon that affects the global climate. ENSO is the most prominent interannual climate variability on Earth with large ecological and societal impacts. The future changes in ENSO are still unclear at present but climatic models reveal that under warming climate, the anomalies of tropical Pacific sea surface temperature (SST) are more El-Niño-like and there will be more central Pacific types, instead of eastern Pacific types. The rainfall responses in the equatorial Pacific are projected to intensify and shift eastward, whereas ENSO SST variability and extreme events are expected to increase under climate change.

Climatic variability affects not only society and its economy, but also the different sectors of agriculture, including the wine industry. A higher frequency of drought events, extreme rainfall, heat waves, and spring–summer frost in different wine-growing areas has been recorded, which has led to increased government spending. What must we consider for future vineyard management in this context of variability? For this, it is essential to increase private investment and fiscal spending on R&D and to broaden the technocratic scientific outlook to a more social vision that allows to diversify the scientific discussion. Academic analysis is limited in terms of mostly considering the research developed in countries with greater investment in science. Latin American countries have valued the Spanish–Creole viticultural knowledge over the French and Anglo–Saxon paradigm, being able to propose mitigation and sustainable alternatives to climate change.

This Research Topic was created to propose new approaches that should be considered in the vineyard of the future. The Research Topic includes six original research studies that cover (i) the response of plant materials to drought and thermal stress; (ii) organic management to mitigate greenhouse gases and to preserve soil fertility; and (iii) the development of new technologies for efficient vineyard management.

The broad genetic diversity of vines is vital for adapting viticulture to global warming, and research groups from Israel and France unraveled this subject. Gashu et al. examined 20 grapevine varieties over three seasons, including lesser-known ones like Argaman, Dolcetto, Ruby Cabernet, and Tinto Cão. Notably, the Israeli variety Argaman had the highest petunidin-3-glc and malvidin-3-glc contents. The study revealed significant genetic diversity in phenylpropanoid metabolism among varieties, with Tempranillo being the most susceptible. de Almeida et al. assessed the drought performance of fungi-tolerant grapevine genotypes under water-deficit conditions. The results indicated that genotypic traits significantly influenced water use efficiency (WUE), with genotypes 3176N and G14 exhibiting superior WUE due to distinct physiological regulations. These regulations were linked to enhancements in photosynthesis and light-harvesting efficiency, highlighting their potential mechanisms for drought adaptation.

Research groups from Germany and Italy contributed with lines of investigation related to organic vineyard management. Schneider et al. investigated the deep incorporation of organic amendments in the subsoil due to its higher carbon storage potential. The researchers applied a biochar compost substrate and green waste compost before planting Calardis Musqué vines. Results showed that organic amendments slightly affected soil CO_2_, N_2_O, and CH_4_ emissions, whereas annual climatic conditions significantly affected vine vigor and berry parameters. Based on these findings, long-term studies are suggested to observe soil parameter changes over time. Lucchetta et al. investigated the effects of compost application versus mineral fertilization in a vineyard subjected to land terracing before plantation. Organic treatment significantly increased soil organic matter, nutrient availability, and biological fertility over 3 years. Compost enhanced microbial growth and enzyme activity, and shifted microbial communities toward beneficial bacteria, reducing pathogenic fungi and improving vine nutrient uptake, vegetative growth, yield, and grape quality parameters. These findings suggest that compost can restore soil fertility and improve vineyard performance compared to chemical fertilization.

Research groups from Portugal and Germany contributed with lines of investigation related to biomodeling and emerging technologies. Egipto et al. developed a simple model to estimate grapevine canopy conductance (g_c_), using stomatal conductance (g_sw_), leaf area index (LAI), net solar radiation (Rn), and air vapor pressure deficit (VPD). The model effectively predicted g_c_ and vine transpiration, with significant influence from VPD and g_sw_. The model is simpler than the Penman–Monteith method and eliminates the need for complex monitoring. It is effective for vineyard irrigation management in stressful environments. A non-destructive method using a near-infrared spectrometer to predict grape quality parameters like sugar and acid content in various grapevine varieties was also investigated by Cornehl et al. This method was faster and less expensive than traditional sampling and analysis. The models demonstrated high accuracy, with regression coefficients over 93% for sugars and over 73% for acids. The study suggests potential commercial use, such as smartphone integration, with cloud-based data processing to minimize costs and enhance adoption. Future research should explore the models and method’s accuracy across more grape varieties and environmental conditions.

The vineyard of the future must consider the following: (i) drought-tolerant plant material: to consider genotypes, varieties, and rootstocks with high physiological regulation and metabolism to adapt drought and thermal stress; (ii) WUE: to implement prediction models to manage irrigation efficiently under stressful environments; (iii) non-destructive sensors: to use near-infrared spectrometers embedded in devices to monitor vine and grape parameters in real time; (iv) leaf-to-fruit ratio: optimize fruit-to-leaf ratio to maximize fruit carbon accumulation and improve photosynthetic efficiency; (v) canopy and root structure: improve canopy and root system structure to positively influence WUE and adaptation to variable climatic conditions; (vi) organic amendments: use organic amendments to improve soil fertility and organic matter and enhance the microbial community, thereby improving vineyard health and productivity; and (vii) continuous validation: continually validate models and methods in different grape varieties and environmental conditions to ensure their long-term robustness and accuracy.

